# Muscle atrophy in mechanically-ventilated critically ill children

**DOI:** 10.1371/journal.pone.0207720

**Published:** 2018-12-19

**Authors:** Ryan W. Johnson, Kay W. P. Ng, Alexander R. Dietz, Mary E. Hartman, Jack D. Baty, Nausheen Hasan, Craig M. Zaidman, Michael Shoykhet

**Affiliations:** 1 Department of Pediatrics, Washington University School of Medicine, St. Louis, Missouri, United States of America; 2 Department of Neurology, Washington University School of Medicine, St. Louis, Missouri, United States of America; 3 Department of Medicine, National University, Singapore; 4 Blue Sky Neurology, Englewood, Colorado, United States of America; 5 Division of Biostatistics, Washington University School of Medicine, St. Louis, Missouri, United States of America; 6 St. Louis Children’s Hospital, St. Louis, Missouri, United States of America; 7 Children’s Research Institute, Children’s National Medical Center, Washington, District of Columbia, United States of America; University of Notre Dame Australia, AUSTRALIA

## Abstract

**Importance:**

ICU-acquired muscle atrophy occurs commonly and worsens outcomes in adults. The incidence and severity of muscle atrophy in critically ill children are poorly characterized.

**Objective:**

To determine incidence, severity and risk factors for muscle atrophy in critically ill children.

**Design, setting and participants:**

A single-center, prospective cohort study of 34 children receiving invasive mechanical ventilation for ≥48 hours. Patients 1 week– 18 years old with respiratory failure and without preexisting neuromuscular disease or skeletal trauma were recruited from a tertiary Pediatric Intensive Care Unit (PICU) between June 2015 and May 2016. We used serial bedside ultrasound to assess thickness of the diaphragm, biceps brachii/brachialis, quadriceps femoris and tibialis anterior. Serial electrical impedance myography (EIM) was assessed in children >1 year old. Medical records were abstracted from an electronic database.

**Exposures:**

Respiratory failure requiring endotracheal intubation for ≥48 hours.

**Main outcome and measures:**

The primary outcome was percent change in muscle thickness. Secondary outcomes were changes in EIM-derived fat percentage and “quality”.

**Results:**

Of 34 enrolled patients, 30 completed ≥2 ultrasound assessments with a median interval of 6 (IQR 6–7) days. Mean age was 5.42 years, with 12 infants <1 year (40%) and 18 children >1 year old (60%). In the entire cohort, diaphragm thickness decreased 11.1% (95%CI, -19.7% to -2.52%) between the first two assessments or 2.2%/day. Quadriceps thickness decreased 8.62% (95%CI, -15.7% to -1.54%) or 1.5%/day. Biceps (-1.71%; 95%CI, -8.15% to 4.73%) and tibialis (0.52%; 95%CI, -5.81% to 3.40%) thicknesses did not change. Among the entire cohort, 47% (14/30) experienced diaphragm atrophy (defined *a priori* as ≥10% decrease in thickness). Eighty three percent of patients (25/30) experienced atrophy in ≥1 muscle group, and 47% (14/30)—in ≥2 muscle groups. On multivariate linear regression, increasing age and traumatic brain injury (TBI) were associated with greater muscle loss. EIM revealed increased fat percentage and decreased muscle “quality”.

**Conclusions and relevance:**

In children receiving invasive mechanical ventilation, diaphragm and other skeletal muscle atrophy is common and rapid. Increasing age and TBI may increase severity of limb muscle atrophy. Prospective studies are required to link muscle atrophy to functional outcomes in critically ill children.

## Introduction

Muscle atrophy in critically ill children may influence illness progression and functional recovery, yet it remains understudied. Only three prospective studies to date have examined intensive care-acquired muscle weakness (ICU-AW) or atrophy (ICU-AA) in children [1, 2]. Banwell et al. used neuromuscular exam to identify a 1.7% incidence of weakness among all children admitted to the PICU for >24 hours (n = 830)[1]. Recently, however, Valla et al. used ultrasound to measure quadriceps femoris in critically ill children receiving invasive mechanical ventilation and identified 59% (10/17) with ≥10% decrease in thickness within 5 days of endotracheal intubation [2]. Glau et al. found that diaphragm thickness in children receiving mechanical ventilation decreases on average by 3.4% per day [3]. The two latter studies concur with robust adult data which indicate that ICU-AA, including diaphragm atrophy, and ICU-AW affect 30–70% of patients [4–7]. A comprehensive assessment of muscle wasting and identification of potential risk factors during pediatric critical illness remains lacking.

In particular, critically ill adults receiving mechanical ventilation exhibit diaphragm thinning 24 hours after intubation [8]. Diaphragm thickness may decrease by 3–6% per 24 hours of mechanical ventilation [3, 9]. Diaphragmatic and other skeletal muscle atrophy and weakness are associated with difficulty weaning from mechanical ventilation [10–13], prolonged ICU stay [11–13], worse functional outcomes [14, 15] and increased mortality [16–18]. Specific risk factors identified in critically ill patients include prolonged mechanical ventilation [7, 19], neuromuscular blockade [3], sepsis [20, 21], multi-organ dysfunction syndrome (MODS)[5, 20, 21], and use of glucocorticoids [19].

We examined how muscle thickness and electrical properties change over time in mechanically-ventilated critically ill children. Both muscle thickness [22, 23] and electrical impedance [24, 25] correlate with muscle strength, and thus may predict physical disability and need for rehabilitation after PICU discharge. We used ultrasound to serially measure thickness of the diaphragm, biceps brachii/brachialis, quadriceps femoris and tibialis anterior. These muscles are easily examined with bedside ultrasound equipment routinely available for vascular access. We also performed serial electrical impedance myography (EIM) on limb muscles to examine EIM utility in the PICU. Finally, we examined whether demographic and clinical variables thought to correlate with atrophy in adults influence muscle loss in critically ill children.

## Methods

### Study design

We performed a single-center, prospective, cohort study in a 35-bed medical-surgical PICU at a free-standing, academic, tertiary care children’s hospital (St. Louis Children’s Hospital (SLCH), St. Louis, MO). Washington University School of Medicine (WUSM) physician faculty staffs the SLCH PICU. The WUSM Institutional Review Board approved the study (IRB #201504013). We recruited patients between June 2015 and May 2016. We enrolled 34 children aged 1 week—18 years who satisfied the following enrollment criteria: 1) normal neurologic development and gross motor function if <15 months old or independently ambulatory before hospitalization if >15 months old; 2) no known neuromuscular disease; 3) respiratory failure requiring endotracheal intubation; 4) intubated <72 hrs before enrollment; and 5) expected to remain intubated >48 hrs. We obtained informed consent from parents and/or guardians at enrollment and, whenever feasible, patients’ assent.

The PICU attending physician directed all clinical interventions, including sedation management. Consistent with the current standard of care, continuous neuromuscular blockade is used sparingly and for the shortest duration possible, as dictated by the patient’s clinical status. Sedation protocol for patients on mechanical ventilation generally involves a continuous infusion of fentanyl with sequential additions of either midazolam or dexmedetomidine infusions. Sedation is titrated to a State Behavioral Score (SBS)[26]; SBS goal is specified on daily morning rounds and depends on the patient’s clinical status and clinical trajectory (e.g. worsening, improving, titrating towards extubation). The objective goal is to minimize sedative use and facilitate recovery without impeding ongoing care.

### Muscle measurements

We measured muscle thickness with bedside ultrasound (US) (SonoSite Edge II, FUJIFILM SonoSite Inc., Bothell, WA) using a 13–6 MHz 6 cm linear probe (L25). We also measured electrical impedance using a commercial EIM device (Skulpt AIM, Boston, MA). Thickness was measured in the right diaphragm, biceps brachii/brachialis, quadriceps femoris, and tibialis anterior. For EIM, we examined biceps, quadriceps, and tibialis in children >1 yr of age. In infants <1 yr old, EIM device size limited observations to quadriceps only. EIM could not be performed on the diaphragm. We obtained US and EIM measurements at enrollment and at 5–8 day intervals until PICU discharge. Observers were blinded to prior measurements.

For US reproducibility, we temporarily marked and regularly reinforced the following skin landmarks: diaphragm: lower ribs along anterior axillary line at end expiration; biceps: 2/3 from acromion to antecubital crease; quadriceps: 1/2 from anterior superior iliac spine to patella’s superior edge; tibialis: 1/3 from patella’s inferior edge to lateral malleolus. We measured thickness with limbs at rest without active flexion/resistance. For biceps, quadriceps and tibialis, the US probe was oriented strictly perpendicular to the skin to minimize error. Cross-sectional images were obtained using thick US gel layer without skin compression. Diaphragm thickness was measured at passive exhalation end with US probe oriented longitudinally along the ribs. We aimed for a simple and rapid assessment, which, in clinical practice, would permit a minimally trained PICU practitioner to obtain reliable measurements without interfering with ongoing care. Consequently, we did not measure diaphragm thickness at end-inspiration, precluding calculation of the thickening fraction[3]. Measurements were obtained in triplicate and averaged for analyses. Intra-rater reproducibility coefficient (*κ*) was 0.99 for biceps, quadriceps and tibialis, and 0.8 for diaphragm, supporting recent suggestions by our group and others that serial US may reliably assess muscle properties in children[2, 27, 28].

For EIM, we used a commercial handheld device (Skulpt AIM, Boston, MA). Per manufacturer’s instructions, EIM device was applied to moistened skin overlying the target muscle with all surface electrodes contacting the skin. As with thickness measurements, we measured EIM while avoiding active flexion/resistance. The EIM device uses proprietary algorithms to report two scores: muscle “quality” and “fat percentage”. Higher muscle “quality” score and lower “fat percentage” score are considered desirable outcomes.

### Outcomes

Primary outcome measure was percent change in thickness between the 1^st^ and 2^nd^ US exams. For patients with >2 US exams, the measurement with the greatest difference from baseline (either increase or decrease) was used. Secondary outcomes were changes in EIM-derived scores. Change in thickness was expressed both in absolute terms and as percent change, because muscle size in children changes substantially during development. In addition, we calculated percent change/day in order to normalize the data for different time intervals between US exams; this normalization does not imply that the change occurs linearly over time. EIM-derived scores–muscle “quality” and “fat percentage”–were compared using change in absolute values.

### Clinical covariates

Demographic and clinical variables that may influence incidence and severity of muscle loss in critically ill children were defined *a priori* and abstracted from the electronic medical record. Non-modifiable variables included age (years), age group (infants <1 yr old *vs* children >1 yr old), PRISM score, SOFA scores on days 1 and 7, traumatic brain injury (yes/no), sepsis as recorded in the medical record by the treating physician (yes/no), PICU and hospital length of stay (days), ventilator-free days at 28 days, body mass index (BMI), Down’s Syndrome (yes/no) and extubation failure. Potentially modifiable variables included glucocorticoids (total dose expressed as hydrocortisone equivalent/kg), neuromuscular blockade (none, single dose, or continuous), hyperglycemia (serum glucose >200 mg/dL), and vasopressors/inotropes (yes/no). We also calculated the Pediatric Risk of Mortality (PRISM) III[29] and daily Sequential Organ Failure Assessment (SOFA) scores [30, 31]. For each organ system, a SOFA organ-specific score >2 defined failure. We defined multi-organ failure as failure of ≥2 systems.

### Statistical analyses

Statistical analyses were performed with SAS Statistics, v 9.4 (SAS Institute Inc., Cary, NC). Kolmogorov-Smirnov test was used to assess normality for continuous variables. Normally distributed variables were compared to using paired Student’s *t*-test. Non-normally distributed variables were compared using Wilcoxon Signed-Rank Test (WSRT). Bivariate relationships between patient characteristics (including risk factors) and continuous outcome variables were examined using simple linear regression. Violations of linear regression assumptions (linearity, multivariate normality, no multicollinearity, no auto-correlation and homoscedasticity) were verified before interpretation of the model. Statistical significance level was set at 0.05. Variables with a *p value* ≤0.20 in bivariate analyses were considered for multivariate model building. In the final multivariate model, *p* ≤0.05 was considered statistically significant.

We defined muscle atrophy *a priori* as ≥10% decrease in thickness. We chose this cut-off because in prior studies, critically ill adults lost ~10% of their muscle mass within one week of hospitalization[32, 33]. Therefore, we additionally examined presence or absence of atrophy in each muscle group as a dichotomous outcome variable. We used bivariate and multivariate logistic regression to assess the relationship between patient characteristics and muscle atrophy. Similar to thickness analyses, risk factors for atrophy with a *p value* ≤0.20 on bivariate analyses were entered into multivariate logistic regression. Results are reported as mean (95% CI) for normally distributed variables and as median (interquartile range (IQR)) for non-normally distributed variables.

## Results

### Participants

**Fig 1:**
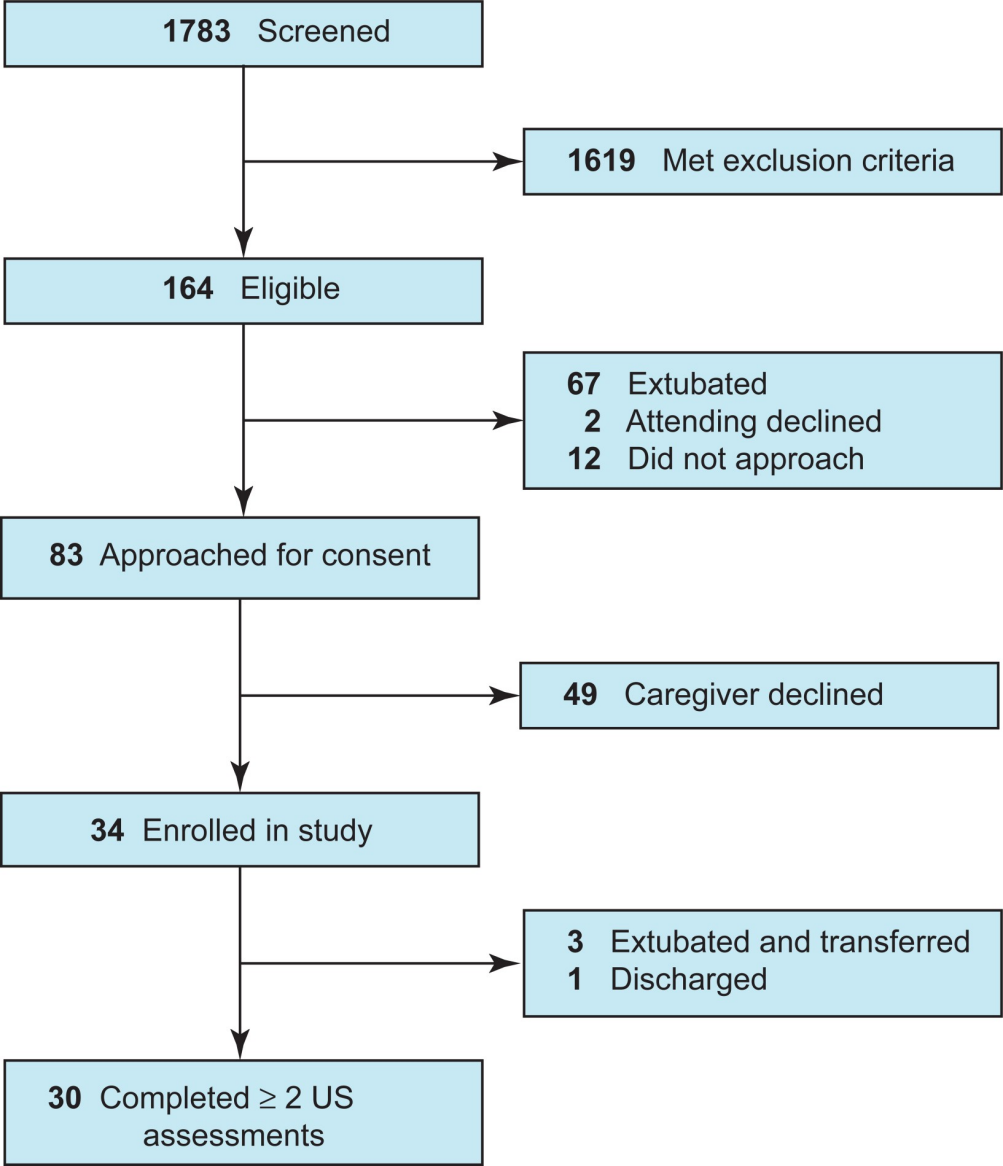
Flowchart of the study population.

We enrolled 34 patients between June 2015 and May 2016 (Fig 1). Thirty patients had ≥2 assessments a median of 6 (IQR 6–7) days apart. Six patients had three assessments. Four patients were transferred (n = 3) or discharged (n = 1) before the second assessment and were excluded from final analyses. Table 1 shows pertinent patient characteristics. The mean age was 5.42 years. Twelve (40%) were infants <1 yr old, and 18 (60%) were children >1 yr old. The most common primary diagnoses were airway/respiratory (33%), central nervous system (24%) and trauma (15%). Mean PRISM III score was 14.72, and mean day 1 SOFA score was 7.85.

**Table 1:** Baseline characteristics of study participants.

Descriptor	All patients (N = 33*)
Age, mean (95% CI), years	5.42 (3.44–7.40)
Age Group, No. (%): Infant (< 1 year)	14 (42)
Child (>1 year)	19 (58)
Female, No. (%)	15 (45)
Race, No. (%): White	26 (79)
Black	4 (12)
Unknown	3 (9)
Body Mass Index, mean (95% CI), kg/m^2^	17.23 (15.76–18.70)
Hospital length of stay, median (range), days	22.10 (5.20–119.50)
PICU length of stay, median (range), days	10.00 (3.3–33.70)
Duration of intubation, median (range), days	7.37 (2.36–22.78)
PRISM III score, mean (95% CI)	14.72 (4.00–30.00)
SOFA score day 1, mean (95% CI)	7.85 (6.51–9.19)
SOFA score day 7, mean (95% CI)	6.61 (5.42–7.79)
Neuromuscular blockade, No. (%): Single dose#	17 (52)
Continuous infusion	10 (30)
Cumulative hydrocortisone equivalent dose, median (range), mg/kg	20.20 (0–1000)
Blood glucose level, mean (95% CI), mg/dL	160.3 (129.8–190.9)
Cumulative insulin dose, median (range), IU	79.01 (0–134.58)
Admission diagnosis, No. (%): Airway/respiratory	11 (33)
Central nervous system	8 (24)
Trauma	5 (15)
Infection/sepsis	3 (9)
Hematology/Oncology	3 (9)
Abdominal	2 (6)
Other	1 (3)

* One patient was discharged home prior to second assessment and was excluded from analyses.

# In addition to the dose used for intubation

At least two US examinations of diaphragm, biceps, quadriceps and tibialis were completed in 100%, 83%, 100% and 97% of patients, respectively (Table 2). The two most significant factors limiting assessment were 1) presence of peripherally-inserted central catheters (PICCs) which limited access to biceps landmarks and 2) liberation from mechanical ventilation and associated sedation in infants prior to second assessment, which precluded passive extension of the extremities. Two EIM assessments of biceps, quadriceps and tibialis were completed in 50%, 70% and 53% of patients, respectively (Table 2). The main factor limiting EIM device use was patient size. Among infants <1 yr old, we successfully conducted quadriceps EIM in only 4 subjects. Among children >1 yr old, however, we completed two EIM assessments of biceps, quadriceps and tibialis in 83%, 89% and 83% of subjects, respectively (Table 2). Therefore, we limited EIM analyses to children >1 yr old.

**Table 2:** Percent of patients completing at least two assessments.

Muscle Group	Ultrasound			Electrical Impedance Myography		
	All	Infants	Children	All	Infants	Children
Diaphragm	100% (30/30)	100% (12/12)	100% (18/18)			
Biceps	83% (25/30)	75% (9/12)	89% (16/18)	50% (15/30)	0% (0/12)	83% (15/18)
Tibialis	97% (29/30)	92% (11/12)	100% (18/18	53% (16/30)	8% (1/12)	83% (15/18)
Quadriceps	100% (30/30)	100% (12/12)	100% (18/18)	70% (21/30	25% (3/12)	100% (18/18)

### Change in muscle thickness

In the entire cohort, diaphragm thickness decreased 11.1% ([95%CI, -19.7% to -2.52%]; p = 0.013) or by 2.2%/day. Sixteen subjects had their 2^nd^ diaphragm US a median of 1 day after extubation [IQR 1–4] and 14 –either prior to or on the day of extubation [median 3 days prior, IQR 0–7]. The timing of the 2^nd^ US exam relative to extubation did not affect the decrease in diaphragm thickness (WRST, p = 0.44).

**Fig 2:**
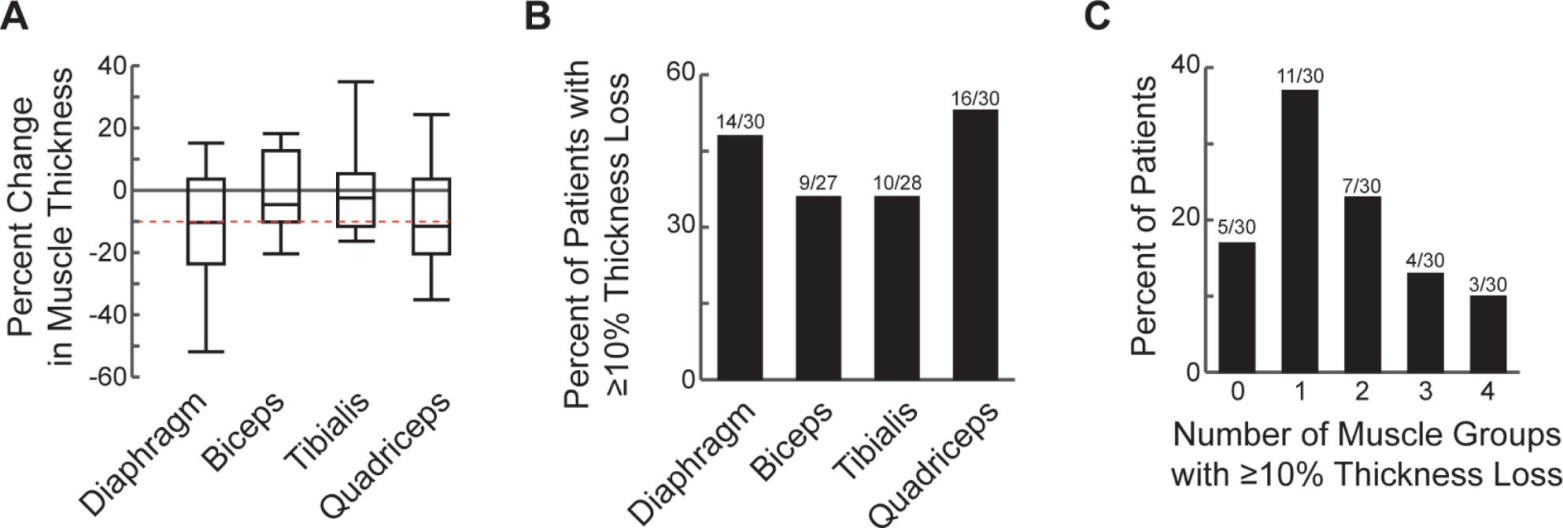
Loss of muscle thickness in critically-ill children. *A*. Percent change in muscle thickness between the first two ultrasound assessments in the entire study cohort. Dashed red line indicates a 10% decrease. Gray line at zero is shown for clarity. Box boundaries represent 25^th^ and 75^th^ percentiles, whiskers– 5^th^ and 95^th^ percentiles. *B*. Percent of patients experiencing muscle atrophy, defined as >10% loss of muscle thickness. *C*. Frequency distribution of patients by the number of muscle groups showing atrophy.

**Fig 3:**
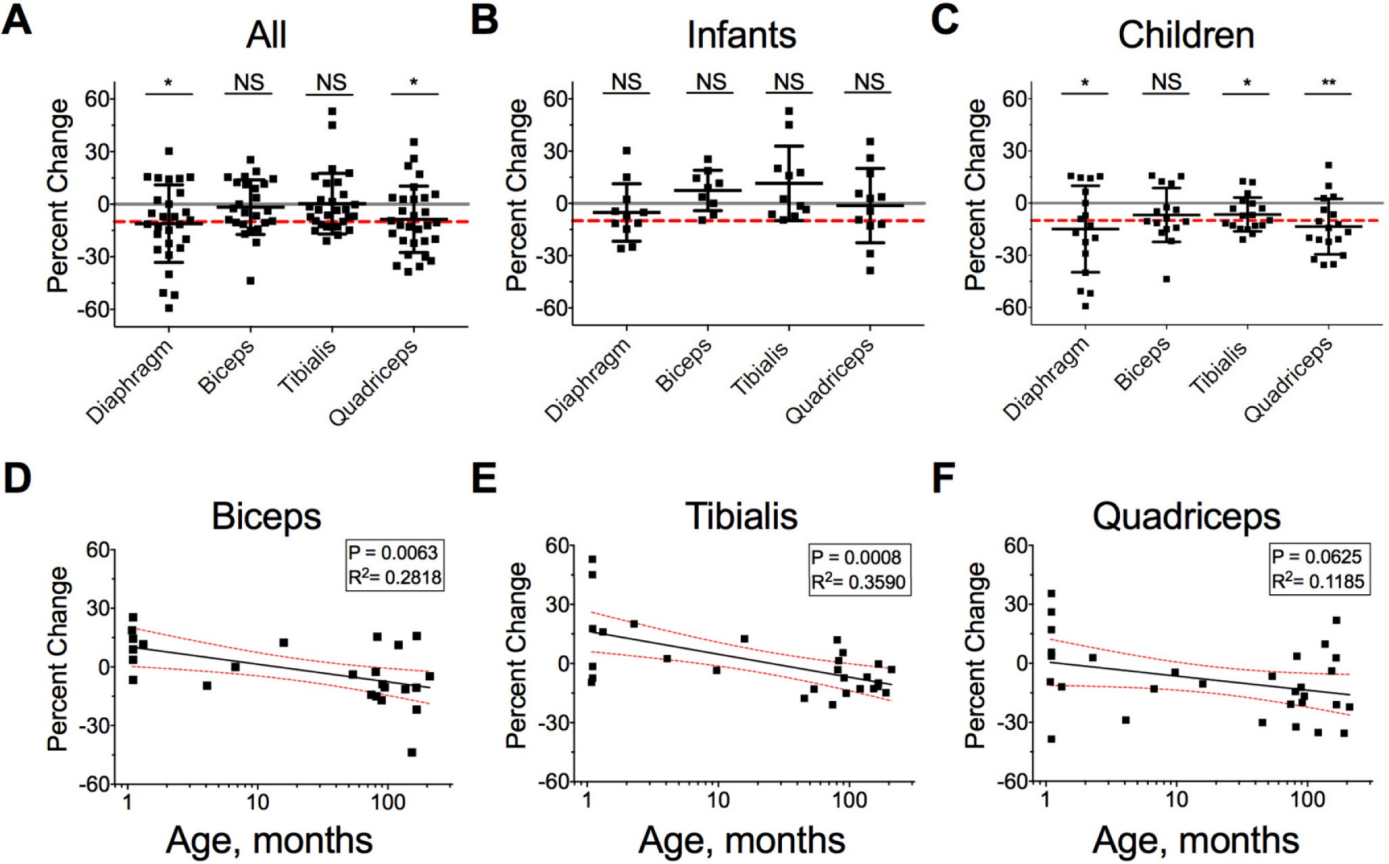
Change in muscle thickness and correlation with age in critically-ill children. *A-C*. Percent change in muscle thickness in the four examined muscle groups in all patients (*A*), infants < 1 yr of age (*B*), and children > 1 yr of age (*C*). Scatter data are shown with mean ± SD. The red line indicates a 10% decrease in thickness. The gray line indicates zero for convenience. *WSRT p < 0.05. *D-F*. Simple linear regression of percent change in muscle thickness as a function of age in months for the Biceps (*D*), Tibialis (*E*) and Quadriceps (*F*). Red dashed lines indicate 95% CI for the slope.

Quadriceps thickness decreased 8.62% ([95%CI, -15.7% to -1.54%]; p = 0.0187) or by 1.5%/day. We did not detect a decrease in either biceps (mean = -1.71%; [95%CI, -8.15% to 4.73%]; p = 0.5885) or tibialis (mean = 0.52%; [95%CI, -5.81% to 3.40%]; p = 0.4762) thickness (Fig 2A and Fig 3A). We defined muscle atrophy *a priori* as ≥10% decrease in thickness. Approximately half of all children examined experienced diaphragm (47%, 14/30) or quadriceps (53%, 16/30) atrophy (Fig 2B and Fig 3A). Among the entire cohort, 83% (25/30) experienced atrophy in ≥1 muscle group and 47% (14/30) in ≥2 muscle groups (Fig 2C). Among children >1 yr old with all 4 muscle groups measured at least twice (n = 16), 94% (15/16) experienced atrophy in ≥1 muscle group and 56% (9/16)—in ≥2 muscle groups.

**Fig 4:**
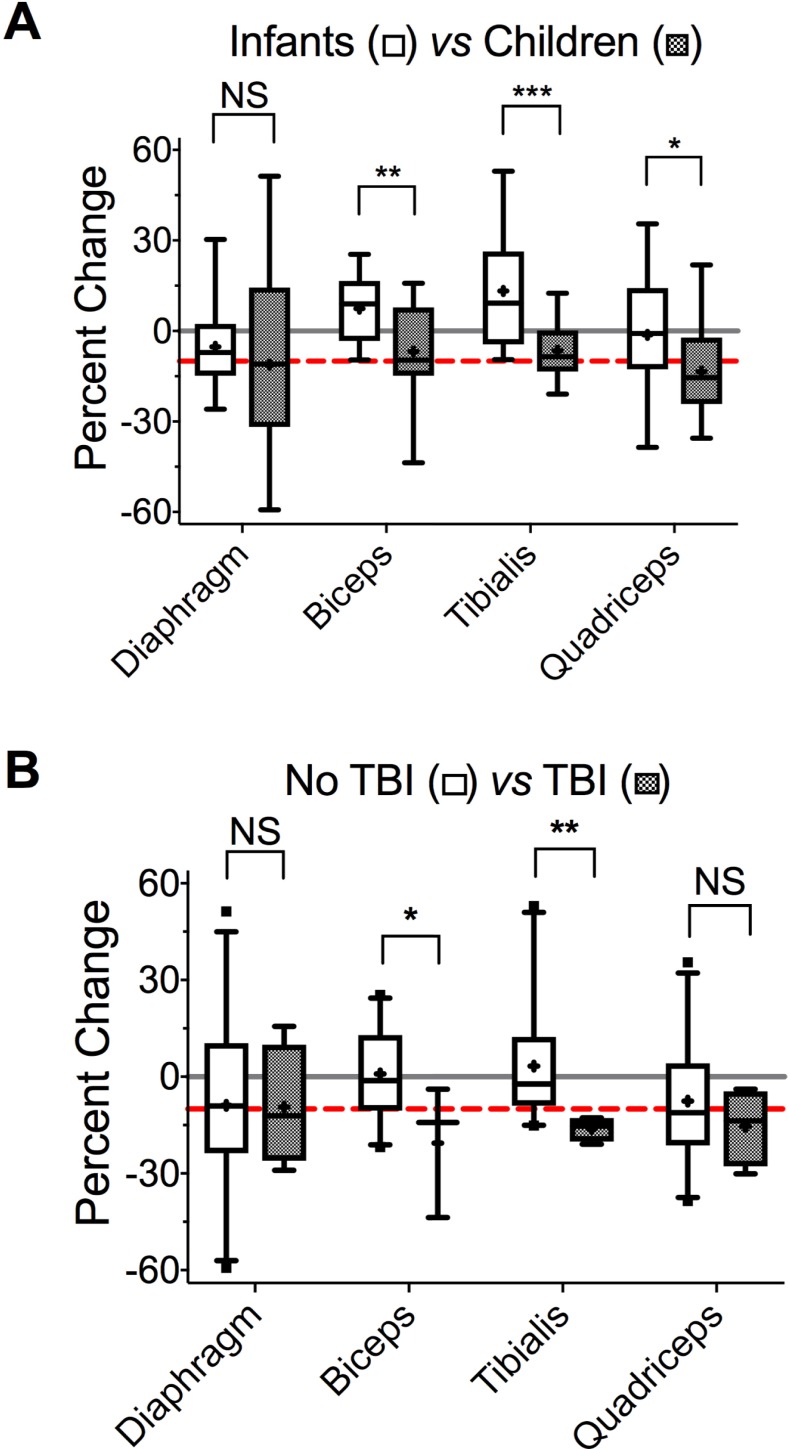
Effect of age group and TBI on change in muscle thickness in critically-ill children. *A*. Change in muscle thickness in infants < 1 yr of age (open) and in children > 1 yr of age (closed). *p = 0.084, **p = 0.025, ***p = 0.002, paired *t*-tests. To account for multiple comparisons, discovery (q) was determined using the two-stage linear step-up procedure of Benjamini, Krieger and Yekuteli (BKY) with FDR = 10%; *q = 0.061, **q = 0.028, ***q = 0.005. *B*. Change in muscle thickness in patients without TBI (open) and with TBI (closed). *p = 0.022, **p = 0.038, *t*-tests. Similar to *A*, discovery was determined with BKY and FDR 10%; * and **q = 0.043. For both *A* and *B*, dashed red line indicates a 10% decrease, and solid gray line–zero. Box boundaries represent 25^th^ and 75^th^ percentiles, whiskers– 5^th^ and 95^th^ percentiles.

We conducted bivariate and multivariate analyses to identify variables associated with muscle loss. Bivariate analyses (Table 3) revealed that increasing age and being a child (*vs*. infant) predicted greater decrease in the thickness of biceps, quadriceps and tibialis but not diaphragm (Fig 3B–3F and Fig 4A). Indeed, children > 1 yr of age on average lost 6.8% of their biceps (95%CI [-14%, 0.8%]), 6.5% of their tibialis (95%CI [-11%, -2%]), and 15% of their quadriceps (95% CI [-22%, -7.2%]) thickness. Increasing BMI correlated with greater loss in biceps and quadriceps muscles (Table 3). Patients with TBI (n = 4) experienced significantly greater decreases in biceps and tibialis thickness than either children or infants without TBI (Fig 4B). On average, children with TBI lost 21% and 17% of biceps and tibialis thickness, respectively. In comparison, children without TBI lost 3.7% and 3.8% of biceps and tibialis thickness, respectively.

**Table 3:** Summary of bivariate associations.

Patient Characteristics	Muscle Group							
Discrete Variables	Diaphragm		Biceps		Tibialis		Quadriceps	
	Median (IQR)	*p*	Median (IQR)	*p*	Median (IQR)	*p*	Median (IQR)	*p*
Sex: Male	-8.1 (-15.9, 15.0)	0.28	-1.2 (-9.6, 11.6)	0.61	-6.3 (-13.0, 5.5)	0.31	**-12.5 (-25.6, -8.0)**	**0.09**
Female	-11.4 (-25.9, 0.0)		-8.8 (-11.5, 14.4)		-3.3 (-7.4, 17.7)		**2.8 (-20.0, 17.0)**	
Age Group: Infant, <1y	-7.1 (-14.8, 2.0)	0.56	**-9.0 (0.0, 14.4)**	**0.03**	**9.24 (-3.5, 20.1)**	**0.01**	**-0.82 (-12.4, 11.3)**	**0.08**
Child, >1y	-11.0 (-29.0, 14.3)		**-9.6 (-14.6, 4.4)**		**-8.6 (-13.0, 0.24)**		**-15.5 (-22.2, -3.90)**	
Race: White	-7.14 (-25.0, 14.3)	0.86	-6.6 (-11.5, 11.6)	0.57	-7.3 (-12.8, 12.0)	0.34	-12.0 (-21.0, 2.9)	0.44
Black	-11.5 (-31.1, -2.4)		0.0 (-15.4, 15.0)		-3.2 (-3.5, 1.5)		-8.7 (-22.4, -0.4)	
Unknown	-2.68 (-20.0, 14.6)		3.6 (-4.8, 15.8)		-0.2 (-3.1, 52.9)		21.8 (-22.2, 35.5)	
Cancer: Yes	-20.0 (-59.3, 51.3)	0.69	-2.5 (-10.4, 15.8)	0.64	-0.2 (-15.1, 12.0)	0.50	-14.3 (-16.7, 21.9)	0.78
No	-8.12 (-22.4, 6.5		-4.3 (-11.5, 11.6)		-3.5 (-11.6, 5.5)		-10.4 (-22.2, 3.6)	
Trisomy 21: Yes	-51.3 (-51.9, -50.6)	0.25	-9.6 (-11.5, 0.0)	0.24	-10.6 (-13.9, -5.0)	0.39	**-9.4 (-20.0, 2.8)**	**0.04**
No	-7.1 (-17.7, 15.0)		0.6 (-9.6, 11.6)		2.5 (-7.4, 16.0)		**-12.1 (-21.6, 4.6)**	
Hyperglycemia: Yes	-14.8 (-29.0, 0.0)	0.25	-9.6 (-11.5, 0.0)	0.32	**-10.6 (-13.9, -5.0)**	**0.05**	-9.4 (-20.0, 2.8)	0.68
No	-7.1 (-17.1, 15.0)		0.6 (-9.6, 11.6)		**2.5 (-7.4, 16.0)**		-12.1 (-21.6, 4.6)	
Asthma: Yes	-11.5 (-11.5, -11.5)	0.84	0 (0.0, 0.0)	0.78	—	—	-12.9 (-12.9, -12.9)	0.81
No	-8.1 (-23.7, 10.4)		-4.3 (-11.0, 12.0)		-3.3 (-12.2, 8.7)		-10.4 (-21.0, 3.6)	
Sepsis: Yes	-20.0 (-22.4, 14.3)	0.66	-3.5 (-11.0, 9.7)	0.50	-3.5 (-11.6, 45.1)	0.55	6.3 (-35.5, 21.9)	0.51
No	-8.1 (-21.0, 4.4)		-3.9 (-104, 11.6)		-3.3 (-12.8, 5.5)		-12.1 (-20.9, -0.5)	
TBI: Yes	-12.1 (-23.1, 4.2)	0.90	**-14.2 (-45, -3.9)**	**0.09**	**-15.3 (-19.3,-12.9)**	**0.01**	-13.7 (-25.4, -5.2)	0.56
No	-9.1 (-22.4, 6.5)		**-1.2 (-10.4, 12.4)**		**-2.3 (-8.5, 12.2)**		-11.2 (-21.0, 3.7)	
NMB: None	-15.0 (-22.4, 0)	0.71	-9.7 (-14.2, -2.5)	0.35	-7.2 (-11.6, 5.5)	0.86	-13.2 (-20.0, 2.8)	0.98
Single	-9.1 (-25.9, 2.3)		7.4 ((-10.4, 14.4)		-4.2 (-12.8, 17.7)		-10.7 (-30.1, 9.8)	
Continuous	-7.1 (-11.5, 14.6)		-3.9 (-9.6, 12.4)		-3.2 (-7.4, 1.4)		-8.5 (-22.2, 3.9)	
Continuous Variables	Correlation Coefficient	*p*	Correlation Coefficient	*p*	Correlation Coefficient	*p*	Correlation Coefficient	*p*
Age (years)	-0.20	0.29	**-0.46**	**0.02**	**-0.50**	**0.01**	**-0.32**	**0.08**
BMI (kg/m^2^)	0.04	0.85	**-0.37**	**0.07**	-0.12	0.54	**-0.25**	**0.18**
Hospital LOS (days)	-0.09	0.63	**0.31**	**0.13**	**-0.34**	**0.07**	-0.09	0.60
PICU LOS (days)	0.05	0.81	0.19	0.38	-0.07	0.73	-0.14	0.48
Duration of intubation (days)	-0.08	0.68	0.26	0.22	-0.03	0.87	0.00	0.98
PRISM III score	0.19	0.34	0.21	0.32	0.26	0.20	**0.38**	**0.04**
SOFA score day 1	-0.10	0.61	0.12	0.57	0.08	0.70	0.03	0.86
SOFA score day 7	0.17	0.37	0.13	0.54	0.18	0.35	0.01	0.93
Glucose most abnormal in 24 hrs (mg/dL)	-0.23	0.24	-0.11	0.62	-0.30	0.13	-0.03	0.87
SaO_2_ most abnormal (%)	-0.09	0.64	0.01	0.97	**-0.42**	**0.03**	0.10	0.61
Max cumulative steroid dose (mg)	-0.08	0.67	0.16	0.43	**-0.35**	**0.07**	-0.22	0.25
Cumulative Hydrocortisone equivalent dose (mg/kg)	0.00	0.98	0.23	0.27	-0.22	0.25	-0.12	0.50

Predictor variables with p ≤0.2 on bivariate analysis (Table 3, bold) were included in the multivariate model. For quadriceps, no statistically significant associations were found on multivariate analysis. For biceps, step-wise multivariate linear regression revealed that increasing age and presence of TBI were associated with greater decrease in thickness (Table 4). Adjusting for TBI, each additional year of age was associated with an additional 1.46% decrease in biceps thickness. Adjusting for age, presence of TBI was associated with an additional 18.1% decrease in biceps thickness. In addition, hospital LOS correlated positively with biceps thickness. For tibialis, increasing age also predicted a greater decrease in thickness. Although the effect of TBI on tibialis thickness was not statistically significant, there was an interaction between TBI and age. Adjusting for TBI, each additional year of age was associated with an additional 1.20% decrease in tibialis thickness. Adjusting for age, presence of TBI was associated with an additional 18.9% decrease in tibialis thickness.

**Table 4:** Summary of multivariate analyses.

Risk Factor	Biceps		Tibialis		Quadriceps	
	Slope Coefficient (95% CI)	*p*	Slope Coefficient (95% CI)	*p*	Slope Coefficient (95% CI)	*p*
Age Group	**-11.1** **(-23.1, 1.0)**	**0.07**	**-18.53** **(-31.6, -5.4)**	**0.01**	-9.0 (-23.0, 5.1)	0.20
TBI	**-21.3** **(-37.8, -4.8)**	**0.01**	-9.9 (-27.9, 8.1)	0.26	—	—
Hospital LOS	**0.27** **(0.05, 0.5)**	**0.02**	-0.15 (-0.42, 0.13)	0.28	—	—
Hyperglycemia	—	—	0.04 (-0.28, 0.37)	0.80	—	—
Downs Syndrome	—	—	—	—	-19.0 (-46.1, 8.1)	0.16
PRISM Score	—	—	—	—	0.9 (-0.20, 1.9)	0.10

### Change in EIM parameters

**Fig 5:**
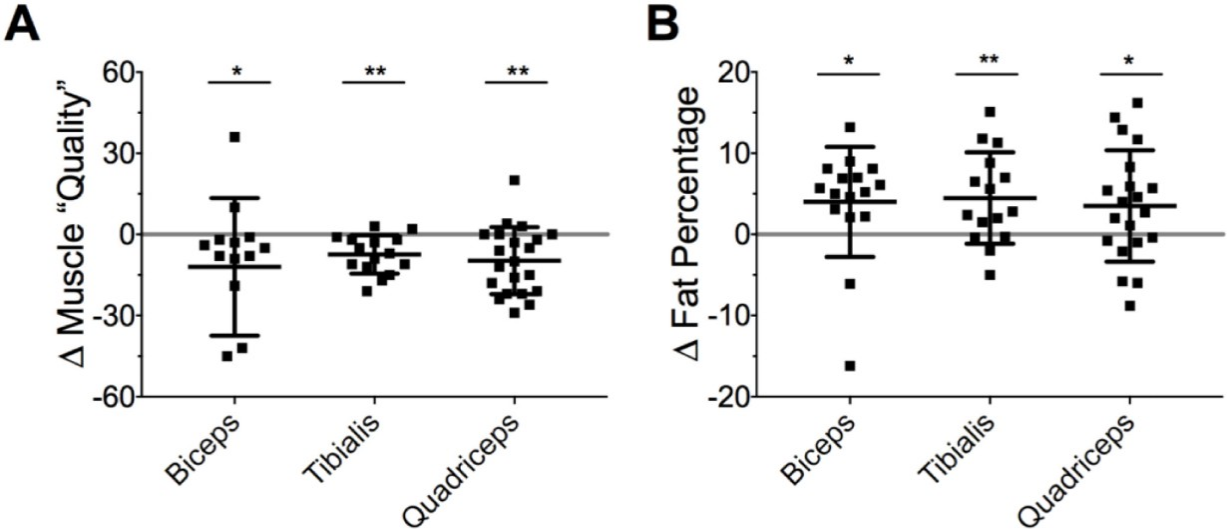
Changes in EIM-derived values in critically-ill children. *A*. Change in muscle “quality” (arbitrary values). *B*. Change in fat percentage. Scatter data are shown with mean ± SD. * p < 0.05, ** p < 0.01, paired *t*-tests.

Muscle “quality” decreased in all muscle groups examined (biceps: - 6.5 [IQR -19 to -2.0], p = 0.040; quadriceps: - 11 [95%CI, -16 to -5.4], p = 0.001; tibialis: -8.1 [95%CI, -12 to -4.1], p = 0.001; all tests WRST; Fig 5A). Fat percentage increased by ~3–5% in all muscle groups examined (biceps: +5.2 [IQR 2.2 to 7.0], n = 14, p = 0.036; quadriceps: +3.5 [95%CI, 0.0 to 7.1], n = 17, p = 0.052; tibialis: +2.8 [IQR -0.3 to 8.8], n = 14, p = 0.009; all tests WRST; Fig 5B).

We also correlated change in EIM parameters and change in thickness. For biceps, increased fat percentage and decreased muscle “quality” correlated with greater decrease in thickness (Spearman ρ = - 0.63, p = 0.017 and Spearman ρ = 0.44, p = 0.11, respectively). We did not observe an association between EIM parameters and thickness for quadriceps or tibialis. Similar to its effect on thickness, TBI was associated with worsening of EIM parameters. On bivariate analyses, only TBI was associated with significant decrease in quadriceps muscle “quality” (Kruskal-Wallis χ^2^ = 4.40, DF = 1, p = 0.036).

## Discussion

The current study used ultrasound and EIM to measure changes in muscle over time in critically ill children with respiratory failure and correlated the extent of muscle loss with potential risk factors. Results indicate that muscle atrophy (≥10% decrease in thickness) in this population is common and rapid, occurring within 5–7 days. Eighty three percent of children experienced atrophy in ≥1 muscle group, and 47% − in ≥2 muscle groups. Importantly, in our study population of critically ill children receiving invasive mechanical ventilation for >2 days, diaphragm atrophy affected almost half. We also found that increasing age and presence of TBI appear to increase muscle loss severity.

Study limitations include single-center nature of the study and relatively small sample size, which may hamper generalization to other centers. Additionally, infants in our PICU do not represent the entire population of infants receiving critical care, as pre-term and full-term infants in the neonatal ICU and infants with congenital heart disease in the cardiac ICU were not included. The SLCH PICU, however, is a tertiary care referral center. Patient population mix and acuity are representative of other large, multidisciplinary PICU’s. One of the inclusion criteria–the requirement that patients were considered likely to remain intubated for at least 48 hours after enrollment–led to selection of a sicker patient cohort than the general PICU population. Consistent with selecting a subset of sicker patients, the median duration of intubation in the current sample is 5 days longer than that in our PICU population as a whole (7.4 *vs*. 2.4 days). Another limitation is lack of long-term follow-up, preventing characterization of functional deficits and/or recovery. The study was designed to investigate the incidence of muscle wasting in critically ill children and to identify potential risk factors. These data are required for larger observational or interventional studies to correlate muscle loss with long-term outcomes. Finally, we used a commercial rather than a research EIM device in an attempt to provide an easy bedside tool for the clinician. The commercial device limited available information to derived values, and size discrepancy between the EIM device and infants’ limbs precluded EIM in infants. Although US data suggest that loss of limb muscle mass may not be a major feature of muscle wasting in critically-ill infants <1 year of age, it is possible that EIM-derived measures would have revealed additional abnormalities.

Our data suggest that diaphragm atrophy in intubated critically ill children occurs rapidly and irrespective of age. Similarly, in critically ill adults, diaphragm atrophy occurs within days[8, 9, 13], and perhaps hours[8], of initiating invasive mechanical ventilation. In animal studies, specific force generated by diaphragm muscle fibers (maximum force normalized to fiber cross-sectional area) decreases 25% six hours into neuromuscular blockade and mechanical ventilation[34]. Diaphragm atrophy in adults predicts difficulty separating from the ventilator, lengthened ICU stay, worse functional outcomes and mortality^9-17^. Similar studies in children do not yet exist, although a retrospective analysis of a large database revealed that ICU-AW was associated with longer mechanical ventilation and with increased risk of discharge to a chronic care or rehabilitation facility[35]. Our data indicate that future studies in children may need to investigate prospectively how diaphragm atrophy affects outcomes such as respiratory muscle strength, duration of mechanical ventilation and extubation failure. Indeed, a recent observational study showed that children with diminished respiratory muscle strength, as evidenced by lower airway pressure generated during airway occlusion, were significantly more likely to require reintubation within 48 hours of extubation[36]. In addition, mechanical ventilation modes that require diaphragm contraction (e.g. Neurally Adjusted Ventilatory Assist, NAVA) may prevent diaphragm atrophy.

In limb skeletal muscles, muscle atrophy in critically ill children appears to depend on age. On average, children >1 year old lost muscle mass in arms and legs, whereas infants <1 year old did not. Indeed, 89% of older children (16/18) lost thickness in ≥1 limb muscle. In contrast, only 41% of infants (5/12) lost thickness in limb muscles. US may be less likely to detect limb muscle loss in infants due to smaller muscle size. Age, however, has been noted as a potential factor in ICU-AW previously. Banwell et al. noted that 11/14 patients with muscle weakness in their cohort were >10 years old, and none was <18 months[1]. One possible explanation is that older children, unlike infants, bear weight. Hence, supine positioning during mechanical ventilation may suddenly decrease limb muscle loads in older children but not in infants.

Muscles atrophied more in children with TBI in our small sample. The association remained significant when adjusted for age. TBI often results in a hypermetabolic state with negative nitrogen balance[37] (although see Mtaweh et al.[38]), which may contribute to exaggerated muscle breakdown. In animal studies, TBI-associated muscle wasting occurs regardless of nutritional status, suggesting that brain injury may initiate specific signaling cascades that alter muscle function[39, 40]. Experimental TBI increased expression of atrophy markers atrogin-1 and m-calpain and altered muscle contractile properties[41]. TBI may also decrease resting muscle tone early after injury, which may contribute to muscle wasting. Our sample of children with TBI is small, and our findings require confirmation in a larger study. If confirmed, the finding that TBI increases severity of acute muscle loss may highlight the need for early interventions to prevent secondary deterioration and improve functional outcomes.

We included EIM to assess its utility in predicting muscle atrophy in critically ill children. EIM is noninvasive and requires minimal training and patient cooperation[42]. Our data show that EIM, as assessed with a commercial device, is feasible in older children but not in infants. Both markers of muscle “quality” and fat percentage deteriorated during the study. The correlation with thickness, however, is relatively weak. It is unknown whether EIM predicts functional outcomes after critical illness. Interestingly, intermittent electrical muscle stimulation may prevent muscle atrophy in critically ill adults[43, 44]. It remains to be determined whether EIM could predict the efficacy of such intervention in critical illness.

The current findings generate several questions and provide impetus for future studies of acute muscle loss in critically ill children. First, does diaphragm atrophy predict difficulty weaning from mechanical ventilation? Second, do mechanical ventilation modes that require sustained diaphragm activity during inspiration prevent diaphragm atrophy? Third, does atrophy severity correlate with functional disability after discharge? Fourth, do interventions such as passive and active motion, early mobilization or electrical stimulation preserve limb muscle mass and improve outcomes in older children? Fifth, how does nutrition affect muscle loss? Sixth, does severe TBI predispose children to acute muscle atrophy and, if so, through what mechanism? Future studies to address these questions in critically ill children are needed.
